# miR-23a-3p/SIX1 regulates glucose uptake and proliferation through GLUT3 in head and neck squamous cell carcinomas

**DOI:** 10.7150/jca.30995

**Published:** 2020-02-10

**Authors:** Hongming Wang, Weishuang Xue, Wunyu Ouyang, Xiaoze Jiang, Xuejun Jiang

**Affiliations:** 1 Department of Otolaryngology, The First Affiliated Hospital of China Medical University, Shenyang, Liaoning, China.; 2 Department of Neurology, The First Affiliated Hospital of China Medical University, Shenyang, Liaoning, China.

**Keywords:** SIX1, head and neck squamous cell carcinoma, glucose metabolism, GLUT3, miR-23a-3p

## Abstract

SIX1 overexpression has been reported in several cancers. However, its involvement in head and neck squamous cell carcinoma (HNSCC) remains unclear. In this study we investigated the clinical significance and biological roles of SIX1 in HNSCC. SIX1 expression was upregulated in HNSCC and correlated with TNM stage and nodal metastasis. Analysis of TCGA dataset demonstrated that high SIX1 expression correlated with poor patient prognosis. Overexpression of SIX1 in the Fadu cell line upregulated cell proliferation, colony formation, glucose uptake and ATP production. In contrast, SIX1 depletion in the Detroit562 cell line downregulated cell proliferation, colony formation, glucose uptake and ATP production. We analyzed a series of genes involved in glucose metabolism and found that SIX1 overexpression upregulated GLUT3, an important glucose transporter, at both mRNA and protein levels. Using the TRANSFAC database, we found that SIX1 had potential binding sites on the GLUT3 promoter, which was validated by chromatin immunoprecipitation (ChIP) assays. Next, we focused on miR-23a-3p, which could target SIX1 in HNSCC cells. The miR-23a-3p mimic downregulated SIX1 expression while the miR-23a-3p inhibitor upregulated SIX1 expression. The binding of miR-23a-3p to the 3'-UTR of SIX1 was confirmed using the luciferase reporter assay. Analysis of TCGA dataset showed a negative correlation between the miR-23a-3p and SIX1. Furthermore, the miR-23a-3p mimic inhibited cell proliferation, ATP production and glucose uptake, which could be rescued by transfection with the SIX1 plasmid. In summary, our study demonstrated that SIX1 facilitated HNSCC cell growth through regulation of GLUT3 and glucose uptake. miR-23a-3p targeted the SIX1/GLUT3 axis and suppressed glucose uptake and proliferation in HNSCC.

## Introduction

Head and neck squamous cell carcinoma (HNSCC) is the sixth most common cancer worldwide 1. HNSCCs are lethal malignancies with a five-year survival rate of only ~50% 2. Local recurrence and distant metastasis after conventional therapy appear to be major contributing factors for restricted survival of HNSCC patients 3. Therefore, understanding the molecular mechanisms of HNSCC progression would help to improve diagnosis and development of novel therapies.

The sine oculis homeobox (SIX) is a family of evolutionarily conserved transcription factors which are characterized by a nucleic acid recognition domain and the SIX domain 4. The SIX1 gene was first identified as a mammalian homolog of the Drosophila sine oculis gene and is highly conserved in numerous invertebrate and vertebrate species 5. SIX1 is highly expressed and plays critical roles in multiple tissues throughout embryogenesis and development 6-9. In most adult tissue, SIX1 is not highly expressed; however, increased SIX1 expression has been documented in multiple cancers, including breast cancer 10, 11, ovarian cancer 12, Wilm's tumors 13, lung cancer 14, hepatocellular carcinoma 15, 16 and colorectal cancer 17. SIX1 mediates tumor initiation and metastasis by regulating various activities of cancer cells, including cell differentiation 18, proliferation 19, 20, the epithelial-mesenchymal transition (EMT) process 10, 21, responsiveness to apoptotic stimuli 12 and genome stability 22. SIX1 overexpression has been reported to indicate poor prognosis of gliomas 23, breast cancers 24 and osteosarcomas 25. However, the clinical significance and biological roles of SIX1 in HNSCC remain unclear.

MicroRNAs (miRNAs) are small (20-22nt), tissue specific, non-coding RNA molecules which cause mRNA translational inhibition or degradation by binding to complementary target mRNAs 26. The miRNAs are predicted to target over 50% of all human protein-coding genes, enabling them to have numerous regulatory roles in many physiological and developmental processes 27. Global downregulation of miRNA expression is an apparent feature of various human tumors 5, 28, including HNSCC 29. Evidence has indicated that miRNAs regulate malignant progression of HNSCC 30, 31. Considering the critical roles of microRNAs in HNSCC, we speculated that certain miRNAs may affect SIX1 expression in HNSCC.

In the present study, we explored the expression and clinical significance of SIX1 in HNSCC tissues. We performed loss- and gain-of-function assays to investigate the biological effects and possible mechanisms of SIX1 in HNSCCs. We also explored the potential function of miRNA-23a-3p, which could negatively regulate SIX1 in HNSCCs.

## Materials and methods

### Patients and samples

The study protocol was approved by the Institutional Reviewer Board of China Medical University. Samples were collected from patients who were diagnosed with cancer and received surgical operations in the First Affiliated Hospital of China Medical University between January 2012 and December 2017. None of the patients received radiotherapy or chemotherapy before surgical resection. The histological diagnosis and differentiation grade were evaluated from sections stained with hematoxylin and eosin according to the World Health Organization (WHO) classification guidelines.

### Immunohistochemistry

Immunohistochemical staining of the cancer tissue sections was performed on 4μm thick sections of formalin-fixed, paraffin-embedded tumor samples. Tumor samples were dewaxed with xylol, and rehydrated with a graded series of ethanol. Endogenous peroxidase was inhibited using 3% H_2_O_2_ for 5 min. Antigen retrieval was performed with citrate buffer in a pressure cooker for 15 min. Ready-to-use goat serum was used for blocking nonspecific binding. Slides were incubated with the primary antibody against SIX1 (1:200, Sigma-Aldrich, St. Louis, MO, USA) at 4°C overnight. Subsequently, the slides were incubated with biotinylated secondary antibody (ready to use, Elivision plus Polymer HRP; mouse/rabbit IHC Kit, Maixin, Fuzhou, China) at 37°C for 2 h. The staining reaction was performed using the DAB plus kit (Maixin). Counterstaining was carried out using hematoxylin, and the sections were dehydrated in alcohol.

The specimens were viewed randomly by two pathologists randomly. Strong nuclear staining was regarded as positive staining. According to a previous publication, we classified staining intensity into 3 grades 32. The staining intensity was scored as 0 (none), 1 (weak), 2 (moderate/strong). Percentages were scored as 1:1-25%, 2: 26-50% 3: 51-75% and 4: 76-100%. Intensity and percentage scores were multiplied to give a final score of 0-8. SIX1 was designated as low expression: score <4; or high expression (overexpression): score ≥4.

### Cell culture and transfection

Cancer cell lines were obtained from the Cell Bank of Type Culture Collection of Chinese Academy of Science(Shanghai, China). Cells were cultured in RPMI-1640 medium with 10% fetal bovine serum (FBS, Gibco, Gaithersburg, MD, USA), 100μg/mL streptomycin and 10U/mL penicillin in a 70% humidity incubator with the conditions of 5% CO_2_ at 37°C. The empty plasmid (Origene, Austin, TX, USA), plasmid with the SIX1 gene (Origene), non-targeting transfection control siRNA (Ribobio, Guangzhou, China) and SIX1 siRNA (Ribobio) were transfected into cells using Lipofectamine 3000 (Invitrogen, Waltham, MA, USA) according to the manufacturer's instructions. FBS-free medium was replaced by medium with 10% FBS after transfection for 4 h.

### Quantitative RT-PCR

Total RNA from cells was extracted using TRIzol reagent (Thermo Fisher, San Jose, CA, USA) according to the manufacture's instruction. RNA was quantified using a spectrophotometer (NanoDrop 2000c, Thermo Fisher, USA). cDNAs were synthesized using PrimeScript RT Master Mix (TaKaRa, Dalian, China), and the total RNAs were used as the template. RT-PCR was performed using the SYBR Green Master Mix with the ABI 7500 Fast Real-time PCR Instrument. The thermal profiles consisted of the following: 50°C for 2min, 95°C for 2 min, 45 cycles of 95°C for 15 sec and 60°C for 40 sec. β-actin was used as the endogenous control and the fold-change of gene amplification was calculated according to the 2-ΔΔCt Method.

### MTT cell viability assay

Cells (5×10^3^/well) were plated in 96-well plates and cultured for 1, 2, 3, 4 and 5 days respectively at 37°C. 20 μL of 5 mg/mL MTT (3-(4, 5-dimethylthiazol-2-yl)-2, 5-diphenyltetrazolium bromide; Sigma Aldrich, St. Louis, MO, USA) solution was added to each well and incubated for 4 hours at 37°C. The supernatant was removed from each well, and 100μl of DMSO was added to dissolve the formazan crystals. The absorbance was measured at 490 nm. Data were obtained from triplicate wells per condition and were representative of at least three independent experiments.

### Western blot

The total proteins of the cells were extracted using lysis buffer containing 1% protease inhibitor cocktail and phosphatase inhibitor cocktail for 30 min at 4°C. Centrifugation was for 10 min at 4°C, and the supernatants were used as the total protein samples. 10μg protein was separated using 10% SDS-PAGE and transferred to polyvinylidene fluoride (PVDF) membranes. The PVDF membrane was incubated in 10% bovine serum albumin solution to block the nonspecific binding. SIX1 (1:1000, Sigma-Aldrich), GLUT1, GLUT2, GLUT3 (1:800, Abcam, Burlingame, CA, USA) and GAPDH (1:1000, Cell Signaling Technology, Danvers, MA, USA) antibodies were incubated at 4℃ overnight. PVDF membranes were washed with TTBS buffer, and were incubated with horseradish peroxidase conjugated anti-mouse/rabbit IgG (1:1000, Cell Signaling Technology) for 2 h at 37°C. Visualization was performed using ECL (Thermo Fisher, USA) and a DNR BioImaging Systems (DNR, Israel).

### Glucose uptake

Glucose uptake was determined using the 2-NBDG glucose uptake assay kit (Biovision, Milpitas, CA, USA). Assays were performed according to the manufacturer's instruction. Cells (2-5 × 10^4^ cells/well) were seeded 1 day before starting the assay. After 8-12 h, remove the culture medium (10% FBS) was removed, and cells were incubated in 400 μL cell culture medium with 0.5% FBS, at 37°C with 5% CO_2_ for 1 h. The cell culture medium was then removed, and 400 μL of glucose uptake mix was added without disturbing the cells, and the cells were incubated at 37°C with 5% CO_2_ for 30 min. After incubation, the cells were harvested from the plate, kept on ice, washed once with 1 mL ice-cold 1× Analysis Buffer, centrifuged at 400 × *g* for 5 min, and the cell pellet was resuspended in 400 μL of 1× Analysis Buffer, and analyzed by flow cytometry (488 nm excitation laser).

### ATP production assay

ATP production was measured using an ATP assay kit (Abcam). The cells were lysed in 200μL of ATP Assay Buffer, centrifuged at 12,000 rpm for 5 min at 4°C, and the protein was removed from the supernatant using a 10 kD spin column (Thermo Scientific). 5μL of the de-proteinated sample was added to the ATP reaction mix in 96-well plates and a fluorescence reading at 535/587nm was measured.

### Luciferase reporter Assay

FaDu cells were seeded into 24 well plates until the confluency reached 60%. miR-23a-3p mimics, luciferase reporter and pRLTK vector carrying SIX1 3′-UTR (binding sites: AAUGUGAA) or SIX1 mutated 3′-UTR (binding sites: AAGGGCGA) were co-transfected into the cells. The transfected cells were cultured for another 24 hours in an incubator and then were lysed using 1×passive lysis buffer (Promega, San Luis Obispo, CA, USA) and the lysates were analyzed using the dual-luciferase reporter assay system (Promega). Independent experiments were performed in triplicate.

### Chromatin immunoprecipitation (ChIP)

ChIP assay was performed using the Magna ChIP G Assay Kit (Millipore, Hayward, CA, USA) according to themanufacturer's instructions. Briefly, cellswere cross-linked with 37% formaldehyde, pelleted, and resuspended in lysis buffer. The cells were then sonicated and centrifuged to remove the insoluble material. The supernatants were collected and incubated overnight with indicated antibodies and Protein Gmagnetic beads. The beads were washed, and the precipitated chromatin complexes were collected, purified, and de-crosslinkedat 62ºC for 2 h, followed by incubation at 95ºC for 10 min. The precipitated DNA fragments were quantified using RT-PCR analysis. The primers for ChIP were as follows:

GLUT3 position1forward, 5' GGTGAATTGGAGAAAGTGTAT 3'; GLUT3 position1 reverse, 5' CCACTCTTCTCTCTCCCCGAT3'; GLUT3 positions2forward, 5' AAGTATACCTACCCTTTGCCC3';GLUT3 position 2reverse, 5' AAAACTATGAGGCAGGGAATG3'.

### Statistical analysis

Data was analyzed using SPSS 17.0 version for Windows (SPSS, Chicago, IL, USA). The possible correlations between SIX1 high levels and clinicopathological factors were determined using the Chi-Square test. Data between treated groups and control groups were determined using Student′s t-test. A value of p<0.05 was considered to be statistically significant.

## Results

### 1. SIX1 is overexpressed in HNSCC

**Figure 1:**
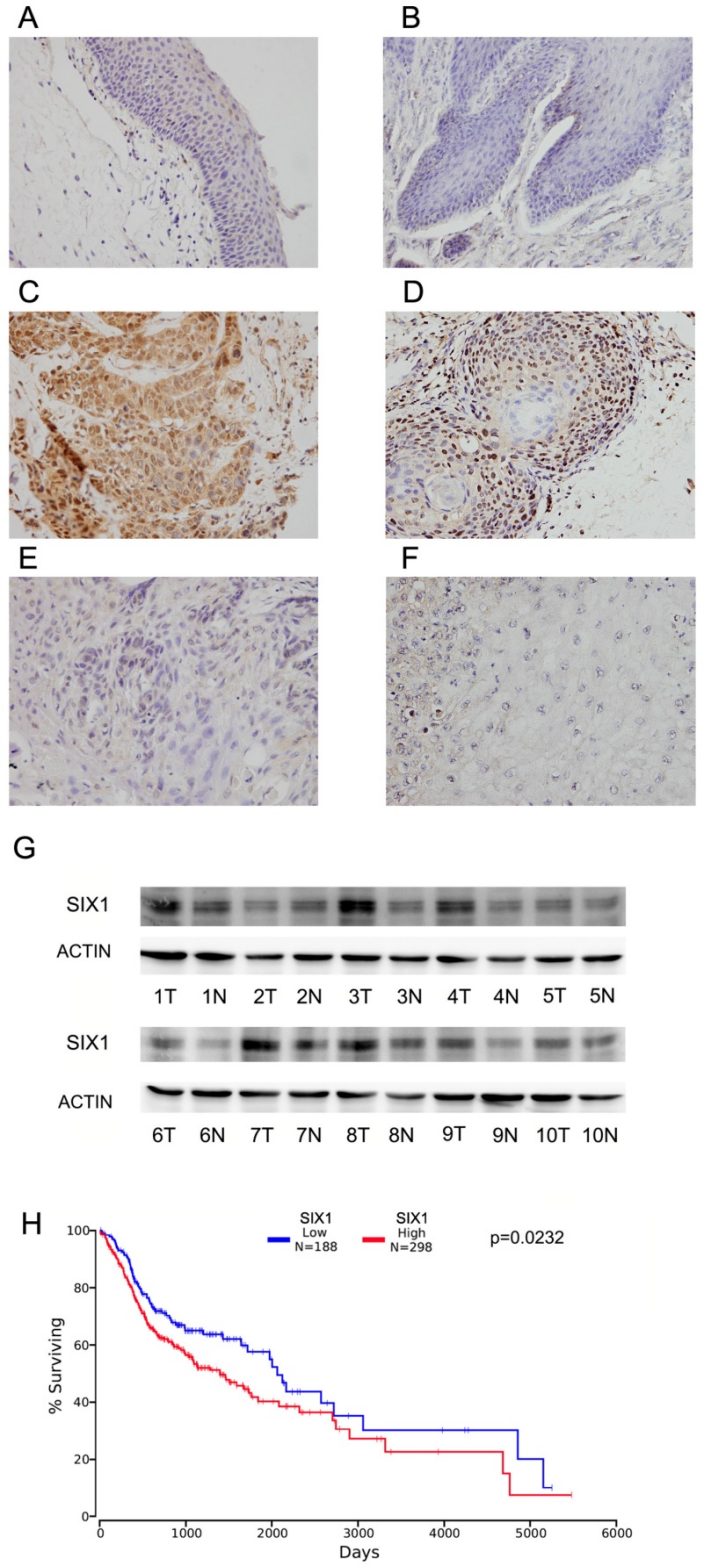
** Expression of SIX1 in HNSCC.** A. Negative SIX1 expression in normal laryngeal squamous epithelium. B. Negative SIX1 expression in a case of normal oral epithelial tissue. C. Positive nuclear and cytoplasmic SIX1 expression in a case of laryngeal squamous cell carcinoma. D. Positive nuclear SIX1 expression a case of oral squamous cell carcinoma. E. Negative SIX1 expression in a case of laryngeal squamous cell carcinoma. F. Negative SIX1 expression in a case of oral squamous cell carcinoma. G. Western blot analysis of SIX1 protein expression in 10 cases of HNSCC tissues and their adjacent normal tissues. H. External datasets from TCGA showed that overexpression of SIX1 correlated with poor overall survival of HNSCC.

Immunohistochemistry of SIX1 was carried out in 102 cases of HNSCC specimens and 10 cases of normal tissues. Nuclear staining of SIX1 was identified as positive staining. Normal epithelial tissues exhibited negative or weak expression (Figure 1A,B), while positive nuclear SIX1 expression were found in 42.1% (43/102) of HNSCC tissues (Figure 1C-F).

We analyzed the correlation between SIX1 overexpression and the clinicopathological factors (Table 1). There was no difference between SIX1 status and age, sex and tumor differentiation. SIX1 overexpression positively correlated with advanced TNM stage (p=0.0002), positive nodal metastasis (0.0297) and advanced T stage (p=0.021).

We also examined SIX1 expression in 10 pairs of HNSCC tissues with their adjacent normal tissues using western blots. We found that SIX1 protein expression was higher in 6 of 10 HNSCC tissues compared with their corresponding normal tissues (Figure 1G).

We then used the The Cancer Genome Atlas (TCGA) database for bioinformatics analyses. RNA-seq data from 496 cases of HNSCC with follow-up information were obtained from TCGA database. High SIX1 status was found to be associated with poor overall patient survival using the Kaplan-Meier plot (*p*=0.0342, log-rank test; Figure 1H). We also performed Cox multivariate analyses using TCGA dataset. As shown in Supplementary Table 1, SIX1 was not an independent predicting factor for poor survival (p=0.0788). These results indicated that high SIX1 expression correlated with HNSCC progression.

### 2. SIX1 regulates proliferation, ATP production, and glucose metabolism in HNSCC cells

**Figure 2:**
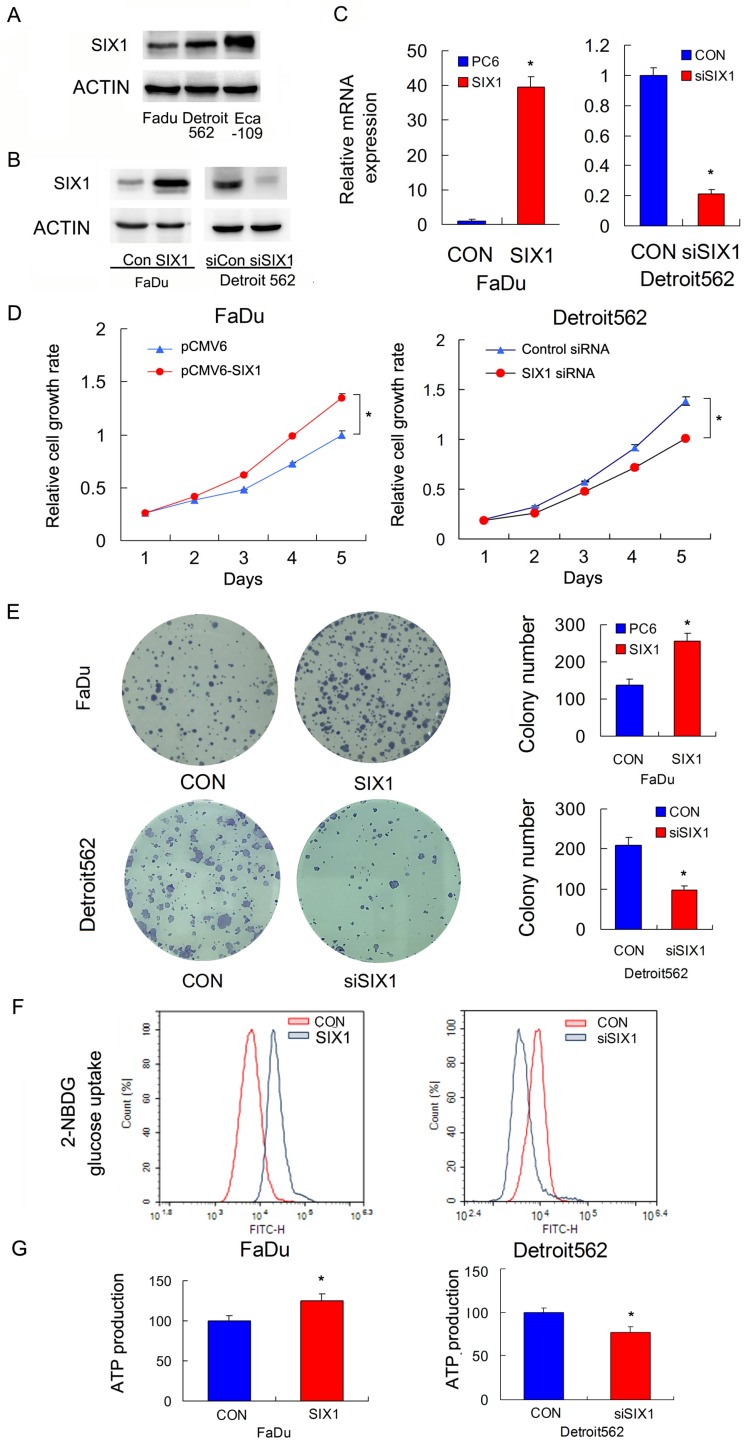
** SIX1 regulates glucose metabolism and ATP production in HNSCC cells.** A. SIX1 protein expression in a 4 HNSCC cell lines. B and C. Efficiencies of SIX1 plasmid transfection in FaDu cell line siRNA knockdown in Detroit562 cell line. D. MTT assay showed that SIX1 overexpression promoted proliferation rate in FaDu cell line. SIX1 depletion inhibited proliferation rate in Detroit562 cell line. E. Colony formation assay demonstrated that SIX1 overexpression upregulated colony number in FaDu cell line while SIX1 depletion downregulated colony number in Detroit562 cell line. F. 2-NBDG glucose uptake assay demonstrated that SIX1 overexpression upregulated glucose uptake in FaDu cell line. SIX1 depletion showed the opposite effect in Detroit562 cell line. G. SIX1 overexpression increased ATP production in FaDu cells while SIX1 depletion decreased ATP production in Detroit562 cells. * indicates p<0.05.

We profiled SIX1 protein expression in a panel of HNSCC cell lines (Figure 2A). The FaDu cell line had relatively low SIX1 expression while the Detroit562 and Eca-109 cell line had relatively high SIX1 expressions. We overexpressed SIX1 in FaDu cells and depleted SIX1 in Detroit562 cells. The transfection efficiency was confirmed by RT-qPCR and western blot (Figure 2B,C). Next the MTT and colony formation assays were conducted to test the effect of SIX1 on proliferation. The MTT assay results showed that the proliferation rate of Detroit562 cells decreased significantly after SIX1 depletion while SIX1 overexpression promoted proliferation rate of FaDu cells (Figure 2D). The colony formation assay results demonstrated that SIX1 positively regulated colony formation ability (Figure 2E).

Glucose uptake is the key step during glucose metabolism and ATP production. We measured glucose uptake using the 2-NBDG incorporation assay and found that SIX1 overexpression significantly increased the glucose uptake rate in FaDu cells while SIX1 depletion decreased the glucose uptake in Detroit562 cells (Figure 2F).These results indicated that SIX1 was as a key regulator of glucose uptake in HNSCC cells.

Cancer cells, including HNSCC, usually adopt aerobic glycolysis of glucose to produce energy (ATP), which is essential for cancer cell survival and malignant growth. To determine if SIX1 regulated metabolism in HNSCC cells, we examined the change of ATP production. As shown in Figure 2G, SIX1 overexpression increased ATP production in FaDu cells while SIX1 depletion decreased ATP production in Detroit562 cells. These data indicated that SIX1 enhanced the rate of metabolism in HNSCC cells.

According to a previous publication, an esophageal cancer cell line Eca-109 have high endogenous SIX1 expression 32. We used it as a positive control cell line and performed siRNA knock down and biological experiments. As shown in Supplementary Figure 1, knockdown of SIX1 effectively downregulated cell growth, glucose uptake and ATP production, suggesting the effect of SIX1 on glucose metabolism was also observed on other types of cancers.

### 3. SIX1 regulates GLUT3 expression in HNSCC

**Figure 3:**
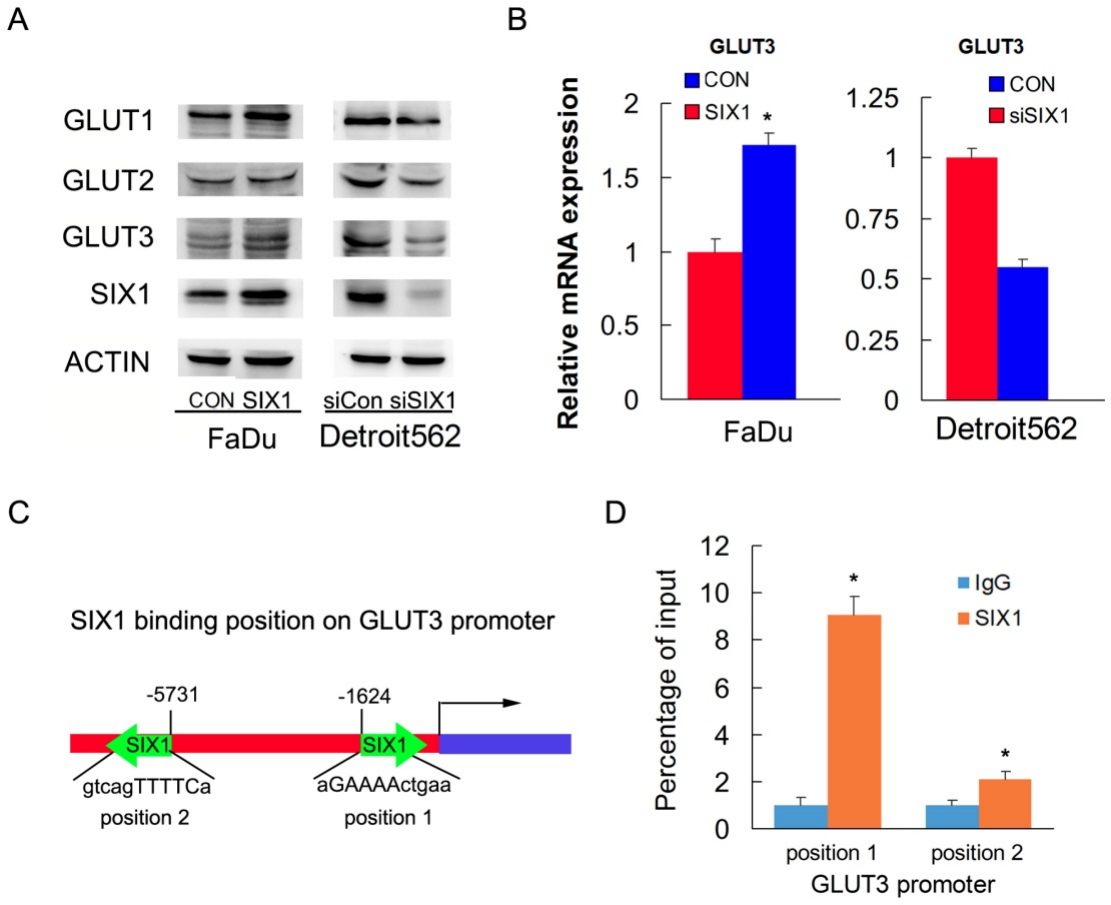
** SIX1 regulates GLUT3 in HNSCC.** A. Western blot showed regulation of SIX1 on GLUT family proteins. B. Real-time PCR shows the effects of SIX1 on GLUT3. C. Prediction of binding sites and sequences by TRANSFAC database analysis. D. ChIP assay showed that SIX1could bind to the GLUT3 promoter. * indicates p<0.05.

To identify the mechanism of SIX1 on glucose uptake, we screened genes related to glucose metabolism using RT-qPCR and western blots. We found that SIX1 overexpression increased protein and mRNA expression of GLUT3 in FaDu cells. In contrast, SIX1 downregulation suppressed protein and mRNA expression of GLUT3 (Figure 3A, B). Because SIX1 has been reported as a transcription regulator, we investigated if SIX1 regulated GLUT3 by binding to its promoter. We used TRANSFAT software to predict the potential binding sites of SIX1 on the GLUT3 promoter (Figure 3C). Binding of SIX1 to the GLUT3 promoter region was validated using chromatin immunoprecipitation (ChIP) assay with anti-SIX1 antibody followed by RT-QPCR assay in FaDu cells (Figure 3D). These results indicated that SIX1 promoted glucose uptake by transcriptional regulation of GLUT3 in HNSCCs.

### 4. miR-23a-3p targets and downregulates SIX1 in HNSCC cells

**Figure 4:**
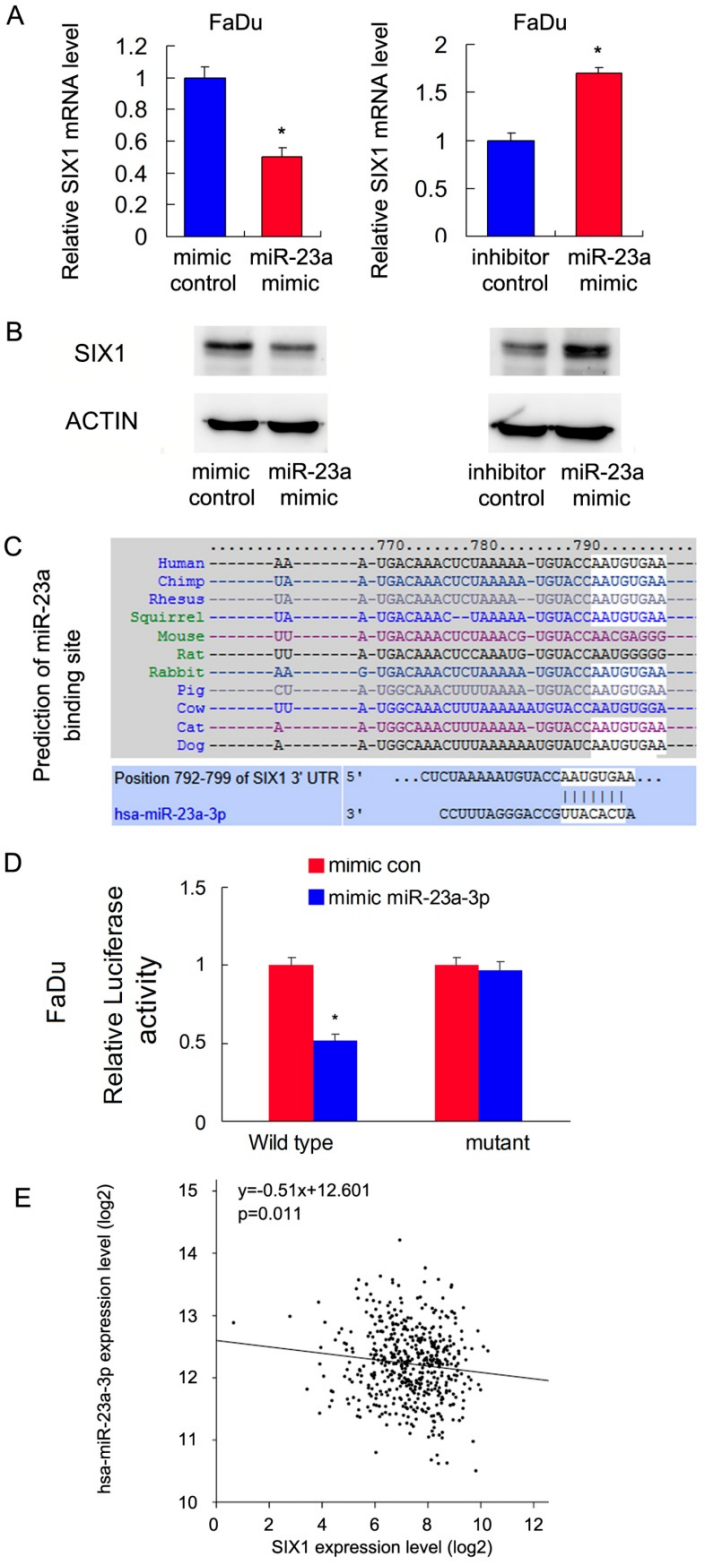
** miR-23a-3p targets and downregulates SIX1 in HNSCC.** A and B. miR-23-3p regulates SIX1 mRNA and protein expression in FaDu cells. C. Prediction of SIX1 as a target of miR-23a-3p using Targetscan analysis. D. miR-23a-3p mimic suppressed the luciferase reporter activity of wild-type reporter, while no significant change was observed in that of mutant reporter. E. TCGA data showed that there was a significant negative correlation between miR-23a and SIX1 expression using linear regression. * indicates p<0.05.

Malignant cells show dependence on dysregulated miRNAs, which in turn control protein coding oncogenes. To identify potential miRNAs that could downregulate SIX1, we used three target prediction software (miRanda, miRwalk and TargetScan). Among the common miRNAs shared by the results from these 3 programs, we mainly focused on miR-23a-3p. We showed that the miR-23a-3p mimic repressed SIX1 expression both at the mRNA and protein levels in FaDu cells. In contrast, the miR-23a-3p inhibitor upregulated SIX1 mRNA and protein expressions (Figure 4 A,B). The putative binding site of miR-23a-3p to the 3'-UTR of SIX1, which was predicted using Targetscan, is shown in Figure 4C. To validate the regulation of miR-23a-3p on SIX1, luciferase reporter assays were performed. The ratio of fluorescence intensity for wild-type and the mutant binding sites was calculated. The miR-23a-3p mimic reduced the luciferase intensity in FaDu cells transfected with vector containing wild-type 3'-UTR, while no significant change was observed in FaDu cells transfected with vector containing mutant binding site, indicating that miR-23a-3p bound to the SIX1 3'-UTR region directly (Figure 4D). To further validate the relationship between miR-23a-3p and SIX1 in HNSCC tissues, we analyzed TCGA data and correlated miR-23a-3p with SIX1 mRNA using linear regression. As shown in Figure 4E, there was a significantly negative correlation between miR-23a-3p and SIX1 expression using linear regression, which further supported our results that miR-23a-3p downregulated SIX1 in HNSCC.

### 5. miR-23a-3p regulates GLUT3 and glucose uptake via SIX1 in HNSCC cells

**Figure 5:**
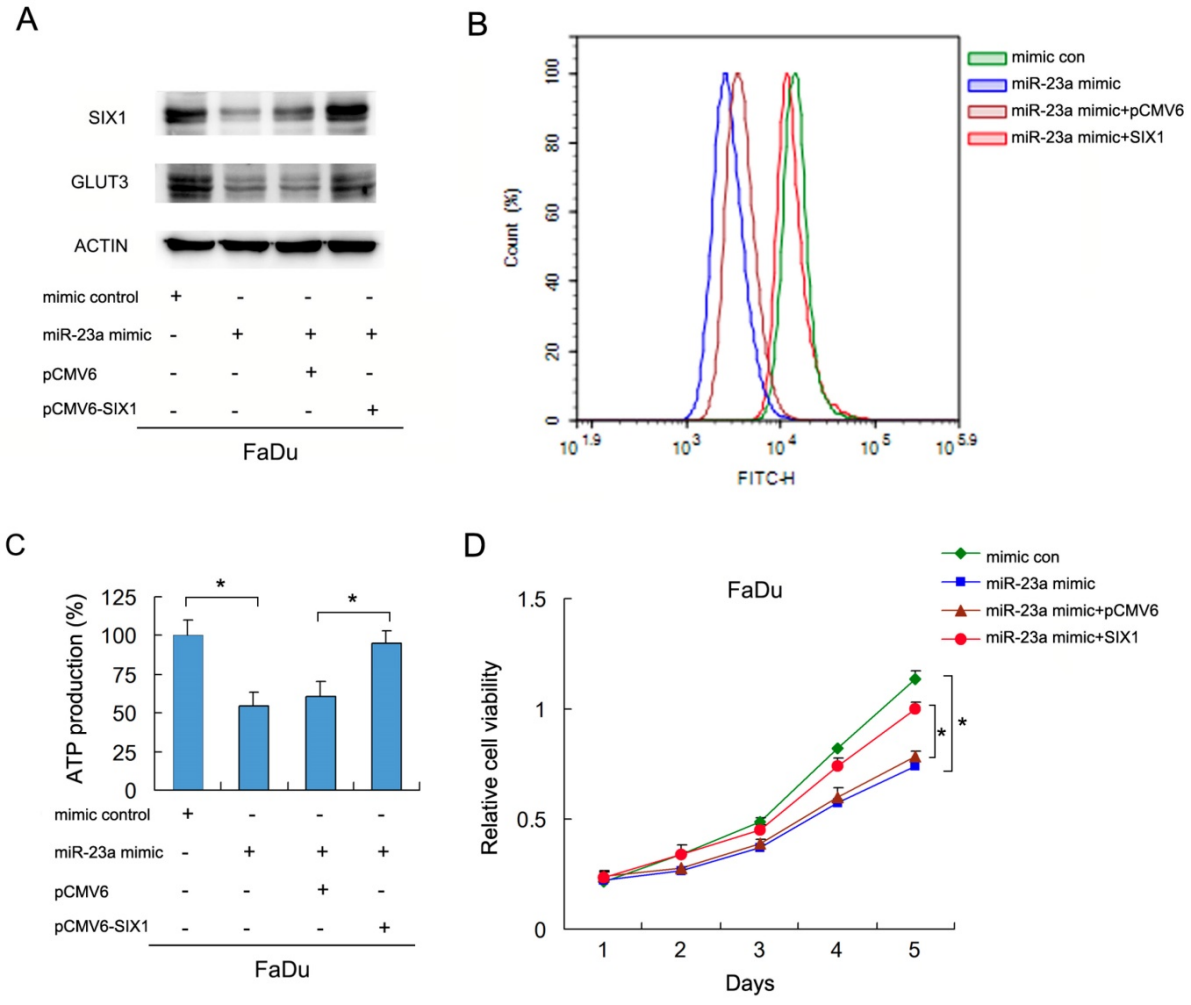
** miR-23a-3p regulates glucose uptake via SIX1 in HNSCC cells.** A. Western blot demonstrated that miR-23a-3p mimic suppressed GLUT3 protein, which was partly restored by SIX1 in FaDu cells. B and C. 2NBDG glucose uptake and ATP production assays showed that miR-23a-3p mimic suppressed glucose uptake, which could be restored by SIX1 overexpression. D. MTT assay showed that miR-23a-3p decreased proliferation of FaDu cells, which was restored by SIX1 overexpression. * indicates p<0.05.

To investigate if miR-23a-3p regulated glucose metabolism in HNSCC cells, we transfected FaDu cells with the miR-23a-3p mimic, with or without SIX1 plasmid. We then examined glucose uptake and GLUT3 expression. As shown in Figure 5A and B, the miR-23a mimic downregulated GLUT3 protein expression. SIX1 partly restored GLUT3 expression in cells transfected with the miR-23a-3p mimic (Figure 5A). The 2-NBDG glucose uptake assay results demonstrated that the miR-23a-3p mimic decreased glucose uptake, which could be restored by SIX1 plasmid transfection (Figure 5B). Analysis of ATP levels also showed that the miR-23a-3p mimic inhibited ATP production, which was rescued by SIX1 overexpression (Figure 5C). MTT assay showed that miR-23a-3p decreased FaDu cell growth, which was restored by SIX1 transfection (Figure 5D). These results indicated that miR-23a-3p regulated glucose metabolism and ATP production by targeting and downregulating SIX1 in HNSCC cells.

## Discussion

Recently, evidence has shown that SIX1 is overexpressed in various cancers including breast, ovarian, colorectal and liver cancer 10-12, 15-17. However, the clinical significance of SIX1 in HNSCC remains unexplored. To address this issue, we checked protein expression patterns of SIX1 and found that SIX1 was overexpressed in HNSCC tissues. High SIX1 status positively correlated with advanced TNM stage and the presence of lymph node metastasis. Importantly, analysis of TCGA data demonstrated that SIX1 overexpression correlated with poor patient survival, making SIX1 a potential prognostic cancer marker in HNSCC.

To explore its biological roles, we examined the role of SIX1 on cell proliferation using the MTT and colony formation assays. Our data demonstrated that SIX1 overexpression promoted cell growth and colony formation ability, while SIX1 depletion showed the opposite effects. Tumor cells tend to shift their metabolism toward aerobic glycolysis, thus producing ATP. Glucose transport is the initial step during glucose metabolism in tumor cells. Here, we found that SIX1 overexpression facilitated glucose uptake into tumor cells and increased ATP production in HNSCC cells, suggesting that SIX1 was a positive regulator of glucose metabolism in HNSCC.

Glucose transporter proteins form membrane transporters that mediate the transport of small carbon compounds across the membranes. GLUT proteins are encoded by the SLC2 genes and are members of the major facilitator superfamily of membrane transporter proteins. In accordance with the high glucose consumption, the GLUT family has been found to be overexpressed in various cancers 33-35. Here, we screened several GLUT family proteins and found that SIX1 mainly upregulated GLUT3 expression at both mRNA and protein levels. According to the prediction of TRANSFAC software, GLUT3 possessed 2 regulatory sites for SIX1, which was confirmed using the ChIP assay. Our results clearly demonstrated that SIX1 was a critical positive regulator of GLUT3 in HNSCC cells. GLUT3 is a highly active glucose transporter, which has been reported as a potential target for anticancer therapy 36, 37. In addition, GLUT3 is overexpressed in HNSCC, which serves as an indicator of poor prognosis 38. Our findings elucidated a novel pathway for GLUT3 upregulation in HNSCC and suggested that a GLUT3 targeting strategy could be achieved by targeting SIX1.

Malignant cells show dependence on the dysregulated miRNAs, which in turn control protein coding oncogenes. These miRNAs provide important opportunities for development of future miRNA-based therapies. Using prediction software, we found that SIX1 was a potential regulator of miR-23a-3p. The biological role of miR-23a-3p has been controversial. Upregulation of miR-23a-3p induced caspase-dependent and -independent apoptosis 39. Overexpression of miR-23a-3p could induced cancer cell hypersensitivity to chemotherapy *in vitro* and *in vivo* 40. In addition, miR-23a-3p has been reported to negatively regulate ATP production and metabolism by targeting mitochondrial glutaminase 41. These reports suggested that miR-23a-3p was a negative regulator of cancer growth and metabolism. In the current study, we confirmed that miR-23a-3p targeted and downregulated SIX1 using RT-qPCR, western blots and the luciferase reporter assay. This was further supported by the analysis of TCGA data showing a negative relationship between SIX1 and miR-23a expression. In addition, our results demonstrated that miR-23a-3p downregulated GLUT3 expression, inhibited glucose uptake, ATP production and HNSCC cell proliferation, which could be rescued by restoration of SIX1 expression. Our data confirmed that miR-23a served as a negative regulator of glucose uptake and metabolism by targeting SIX1/GLUT3 signaling in HNSCC.

In conclusion, our data demonstrated that SIX1 was overexpressed in HNSCC and promoted cancer progression and glucose uptake by transcriptional upregulation of GLUT3. miR-23a downregulated GLUT3 and glucose uptake by targeting SIX1 in HNSCC.

## Supplementary Material

Supplementary figure and table.

## Figures and Tables

**Table 1:** Clinical profile and correlation between the clinicopathological features and SIX1 expression in HNSCC

Characteristics	Number of patients	SIX1 low expression	SIX1 high expression	*P*
Age				
<60	65	38	27	0.8669
≥60	37	21	16	
Gender				
male	72	39	33	0.2441
female	30	20	10	
Differentiation				
Well	58	37	21	0.3081
Moderate	28	13	15	
Poor	16	9	7	
TNM stage				
Ⅰ+Ⅱ	70	49	21	0.0002
Ⅲ+Ⅳ	32	10	22	
Lymph node metastasis				
Absent	77	50	27	0.0297
Present	25	9	16	
Tumor stage (T )				
T1+T2	82	52	30	0.021
T3+T4	20	7	13	
