# Supplementary material for: miR-23a-3p/SIX1 regulates glucose uptake and proliferation through GLUT3 in head and neck squamous cell carcinomas
Source: J Cancer. 2020 Feb 10;11(9):2529–39. doi: 10.7150/jca.30995 (PMC7066005; doi:10.7150/jca.30995)

Supplementary Table 1. COX multivariate analysis for predictive factors (TCGA data)

Factors	Hazard ratio(95% CI)	p value
Age	1.388 (0.996-1.934)	0.0529
Gender	0.781 (0.551-1.107)	0.1646
SIX1	1.350 (0.966-1.886)	0.0788
Tumor grade	1.001 (0.791-1.266)	0.9941
Clinical stage	1.046 (0.879-1.246)	0.6125

Supplementary Figure 1

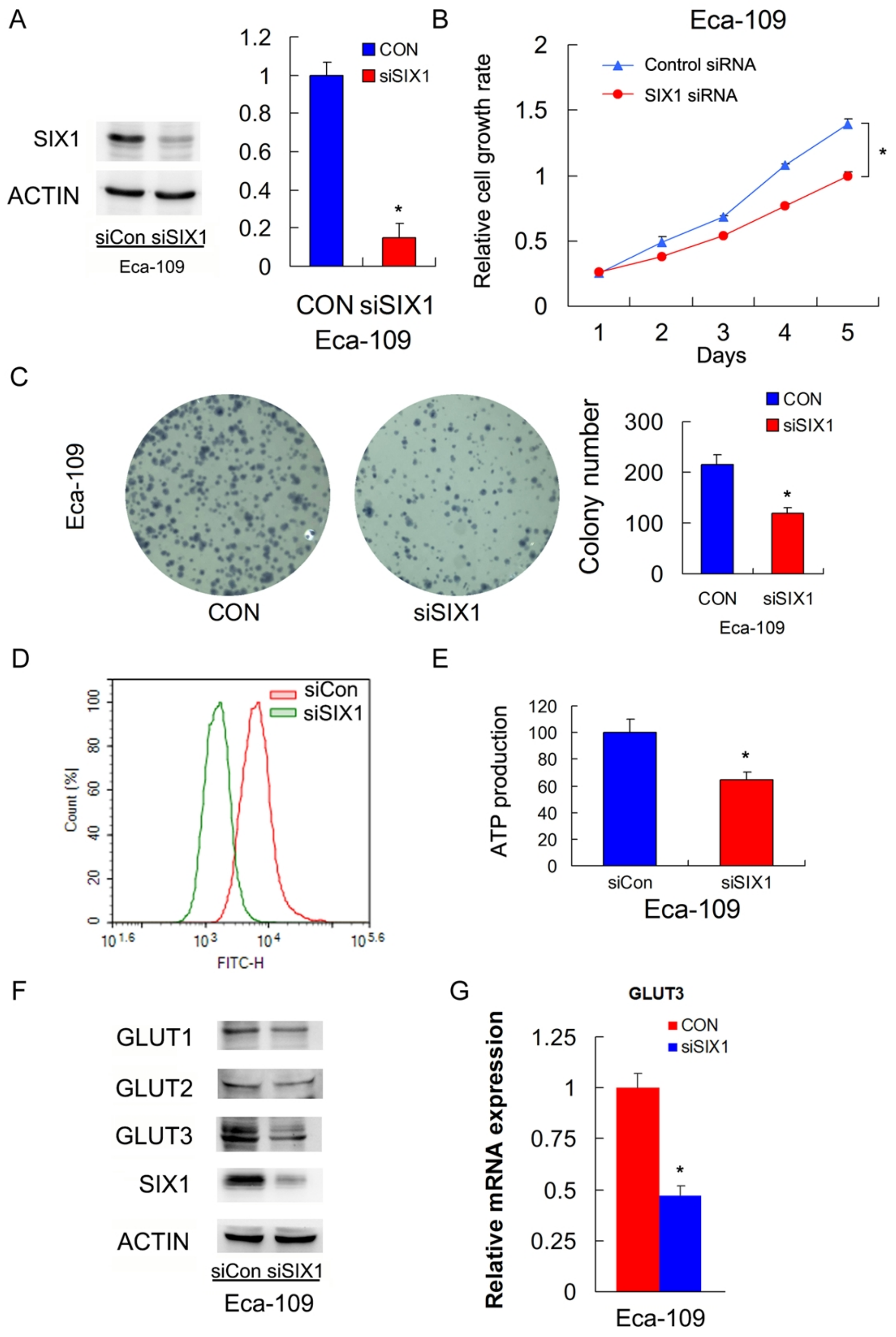

Supplement: Supplementary file 1 — Supplementary figure and table. [file jcav11p2529s1.pdf]
